# Medical Evidence in Cases of Insanity

**Published:** 1851-10-01

**Authors:** 


### ART. V.—MEDICAL EVIDENCE IN CASES OF INSANITY.

THE November number of "*Blackwood's Magazine*" contains an elaborate analysis of the first volume of a work entitled "Modern State Trials."* The analysis in question is said, upon good authority, to be written by Mr. Samuel Warren, the well-known author of the "Diary of a Late Physician," and "Ten Thousand a Year." Of this gentleman's high literary attainments we wish to speak with great respect, but we cannot allow him or any other member of the legal profession, however distinguished may be his position, or varied his attainments, unjustly to attack that section of the medical profession especially called upon to give evidence in our courts of justice in cases of insanity, without entering our formal protest against it. Unfortunately there appears to be but little sympathy and kindly feeling between the members of the *bar* and the professors of *physic*. The aggression is certainly not on our side. We have not been the assailants; the psychologist is solemnly called upon to elucidate in our courts of law points of great intricacy, requiring on his part not only a mind well stored with facts, the result of much experience and observation in the treatment of the insane, but of an understanding fitted by natural organization and training for the ready appreciation of the highest order of metaphysical truth and evidence — viz., that connected with the deviations of the mind from a state of health and responsibility. He is supposed,—and rightly and justly supposed,—by education, study, reflection, and enlarged experience, to be capable of enlightening the court and the jury upon matters with which they are necessarily but superficially acquainted, and he is subpœnaed to give the benefit of his scientific knowledge in cases involving often the questions of LIFE and DEATH. Surely it is the duty of the legal profession if they differ from the views generally propounded by medical men in relation to the plea of insanity to respect their opinions, however opposed they may be to those which they themselves entertain. Sad will be the consequences if, as the results of the injudicious and uncalled for language of the

* By Mr. Townsend, Q.C. This gentleman died shortly after the publication of his work.

Bench, and the raillery and misplaced wit of the Bar, the public should be taught to undervalue or think lightly of, the evidence of the medical psychologist in cases involving the subject of crime and insanity, responsibility and irresponsibility. That such, alas! is the unhappy *tendency* of events we infer from the remarks which are said to have fallen from the lips of the Lord High Chancellor of England, as referred to in an able contemporary.* We quote the following passage:—

"During the recent hearing of a lunacy case, the Lord Chancellor is reported to have said,—'*His experience taught him there were very few cases of insanity in which any good came from the examination of medical men. Their evidence sometimes adorned a case, and gave rise to very agreeable and interesting scientific discussions; but, after all, it had little or no weight with a jury.*' Whatever may be the respect we entertain for this high authority, we emphatically dissent from these views, believing them to be founded in error, and totally unjust to practitioners in medicine, who are not only the most important but often the only competent witnesses, in inquiries respecting insanity. That medical evidence does not always produce the impression it ought upon the individuals addressed, we readily understand. The judicial tribunal just mentioned, from its miscellaneous composition, must be occasionally unable to form correct conclusions respecting the very intricate points at issue; more especially as even great legal authorities themselves are sometimes far from being unanimous in opinion. Suppose an eminent medical professor were to assert, 'ex cathedrâ,' that 'the bar of England knew nothing of law, and that very little benefit ever accrued from their speeches. They might adorn a case, make an eloquent oration, or quote precedents, but all their arguments would never influence any twelve men sitting in a box.' Westminster Hall would be in an uproar against any member of the medical fraternity who presumed to utter such sentiments; and we acknowledge the lawyers might, in that instance, justly express their indignation. Each profession should respect the other, in its peculiar sphere, as all, the public included, will then gain by liberal and kindly bearing. As an illustration of the feelings which ought to prevail, allusion might be made to the late Lord Chief Justice, the son of a physician, and an able as well as dignified jurisconsult, when presiding at the trial of Oxford for treason. On that occasion the present Lord Chancellor, then a learned serjeant, having endeavoured to brow-beat a medical gentleman whilst giving evidence, was properly checked by the presiding judge, in order that the witness need not fear any forensic fervour of counsel. We therefore say to our brethren, when called upon to appear in a court of justice,—state facts and opinions clearly, firmly, respectfully, and fearlessly."

The good sense and proper feeling of the concluding remarks of the above quotation, must be manifest to all. But to the case immediately

* The Lancet, August 2, 1851.

before us. Mr. Warren, after giving a succinct account of the details of M'Naughten's trial, observes as follows:—

"After going through the evidence (if the word can be used with propriety under such circumstances) of the other medical gentlemen, Mr. Townsend observes, 'Each physician and surgeon, as he stepped into the witness-box, seemed anxious to surpass his predecessor in the tone of decision and certainty; each tried to draw the bow of —— (mentioning the first physician who had been called, and who was also called in Oxford's and Pate's case, in which latter he was rebuked by Baron Alderson,) and shoot, if possible, still farther into empty space. And this gentleman, Dr. ——, had asserted, under cross-examination by Sir William Follett, 'his positive conviction that he could ascertain the nicest shade of insanity ! that the shadowy trace of eccentricity, dissolving into madness, could be palpably distinguished!' The last of these confident personages then was permitted to make this extraordinary statement: 'I have not the slightest hesitation in saying that the prisoner is insane, and that he committed the offence in question whilst afflicted with a delusion under which he appears to have been labouring for a considerable length of time ! ! !'"

We wish to speak respectfully of the dead, otherwise we might be disposed somewhat to cavil at the expression, "confident personage," used by Mr. Townsend, particularly as it is applied to the Editor of this Journal. We feel no uneasiness of mind, or compunctions of conscience, in thus "owning the soft impeachment," and admitting that Dr. Winslow is the "confident personage" spoken of in the passage quoted, and that the "extraordinary statement" was made by ourselves. We extract the paragraph containing the offensive epithet, with all its typographical embellishments. But whilst we thus leniently pass over Mr. Townsend's unjustifiable appellation, we are not so disposed to treat Mr. Samuel Warren's *adoption* of the phrase, or his comments upon it. The author of "Ten Thousand a Year," after quoting Mr. Townsend, exclaims, in an affected spirit of honest indignation, "We feel constrained to say that this appears to us, in every way, monstrous." "Monstrous !"—why monstrous, Mr. Warren ? The witness was upon his oath. His mind could not, with any degree of reason, have been supposed to be unduly biassed. He was not a paid witness. He had no connexion either with the prosecuting or the defending party. But being in the court at the time of the trial, he was requested by Sir A. Cockburn to enter into the witness-box, to give his opinion on a point of science; having heard all the evidence, *pro* and *con*, Dr. Winslow was asked, as a person supposed to have some practical knowledge of insanity, to give the jury his opinion of M'Naughten's probable condition of mind when the act, for which he was being tried, was committed ? Dr. Winslow in reply to the question gave the evidence, which Mr. S. Warren, with unpardonable effrontery, stigmatises as "monstrous!" What was the *result* of that evidence? Let Mr. Townsend answer,—

"Chief Justice Tindal here interposed, to ask Sir William Follett whether he was prepared with evidence on the part of the Crown to combat that of the medical witnesses,—

"'Because, if you have not,' said the Chief Justice, 'we think we are under the necessity of stopping the case. Is there any medical evidence on the other side?'

"*Sir William Follett.*—'No, my Lord.'

"*Chief Justice Tindal.*—'We feel the evidence, especially that of the last two medical gentlemen who have been examined, and who are strangers to both sides, and only observers of the case, to be very strong, and sufficient to induce my learned brothers and myself to stop the case.'"*

The evidence of Dr. Winslow did not alarm the discreet, the able, the penetrating, and sagacious mind of the Lord Chief Justice. It was not "monstrous" in his estimation. That distinguished jurist—perhaps the most illustrious judge of modern times—saw in the twinkling of an eye, what Mr. S. Warren, with all his wonderful penetration, cannot after the lapse of years, with the evidence of the trial in detail before him, conjoined with a knowledge of the facts connected with the history of M'Naughten since his confinement in Bethlem for a period of *eight* years, perceive. Lord Chief Justice Tindal saw that the insanity of the prisoner was placed beyond the slightest shadow of a doubt—and such also was the conviction of Dr. Winslow when he gave the "monstrous" evidence alluded to by Mr. Warren. There could be no question as to the fact of M'Naughten's mental derangement. Cases of insanity are sometimes made the subject of investigation in our criminal courts, in which the medical and general evidence is nicely balanced, and when the physician is justified in giving his opinion with extreme caution; but with regard to M'Naughten, the delusions were so evident—so satisfactorily and conclusively established in course of the trial—that a conviction of his total wreck of intellect forced itself upon minds most unwilling to receive it; not only was it proved that M'Naughten was deranged, but that the criminal act sprang directly out of, and was almost the natural and inevitable result or sequence of, the morbid creations of his fancy; thus bringing the question of his responsibility fairly within the test laid down by the celebrated Lord Erskine, in the memorable trial of Hatfield—viz., that insanity should not exempt from punishment when the crime is not *directly traceable to a delusive impression.*

M'Naughten imagined that he was beset by conspirators, and with

* Townsend's Modern State Trials, p. 400.

the view of protecting himself from these imaginary assailants, he carried about his person a loaded pistol—and influenced by this morbid idea, which embittered his very existence, he shot Mr. Drummond. It is a matter altogether distinct from the question at issue, whether he mistook Mr. Drummond for the late Sir Robert Peel. If that distinguished statesman had fallen in lieu of his secretary, it would not, in the slightest degree, have altered our view of the mental aspect of the case. Deeply as we should, in common with the whole world, have deplored the untimely and unhappy death of so great—so illustrious a man—a stern regard for *truth, justice,* and *humanity* would have compelled us to throw the shield of protection around the poor lunatic, deprived by an inscrutable Providence of the right exercise of those faculties given to him for his guidance and self-control.

Continuing his observations on the case, Mr. Townsend remarks:—"Nine medical witnesses had now spoken, with a wonderful unanimity of opinion, *and the court surrendered at discretion.*"

"Surrendered at discretion!" Truth was established; the solemn and sacred claims of justice were vindicated. The able judge then considered it to be his duty to stop the inquiry, so convinced was *he* of the utter uselessness and folly of prolonging so painful an investigation. Did the intelligent jury hesitate? Did they retire from the box apparently unsettled in their minds as to the verdict they ought to return? No sooner had the Lord Chief Justice interfered, than the jury at once bowed to the just decision of Lord Tindal, and without the appearance of the remotest difference of opinion, returned an unanimous verdict of acquittal, on the ground of insanity!

And what says Mr. S. Warren of the effect on the court of the evidence of the "nine medical witnesses?" Hear him?—"If such a course is to be allowed again in a court of justice, what security have any of us for life, liberty, or property?"

Reverse the picture, and what would have been the sad consequences? Would Mr. S. Warren have *executed* M'Naughten? What a spectacle of horror would such a cruel, disgraceful, and barbarous act have been in a Christian country like that in which we are privileged to live! As well might he go to the Highgate Institution for Idiots, and drag out from that abode of lost and ruined minds a poor drivelling, helpless, imbecile child, and inflict upon him corporeal chastisement for a non-comprehension of an honest distinction between *meum* and *tuum*. We do not believe that M'Naughten knew or had the remotest conception that he was "acting contrary to the law of the land;" and if we were convinced that such an idea had passed through his mind immediately before he fired the fatal shot, it would not have altered our opinion of his insanity and total irresponsibility. We do not, as medical psychologists, recognise this as a scientific test of criminal responsibility. The mere consciousness of an act to which an insane person may be prompted, being *contrary to law,* is not *to our minds* a sufficient proof of legal or moral responsibility. Mr. S. Warren thinks differently. So let it be: he is welcome to his opinion.

But what has been the history of M'Naughten since his acquittal and confinement in Bethlem? Mr. S. Warren paid a visit to the Hospital for the purpose of ascertaining his present condition. If his visit had taken place subsequently to his having written the whole of the review of Mr. Townsend's work, we should have looked forward to an article in a future number of *Blackwood,* recanting the opinions expressed in the communication before us. Our readers will participate in our surprise when they are informed that the formal examination of M'Naughten (the details of which we shall presently give) was made *prior* to the completion of the article in question. This certainly appears to us to be most extraordinary. If Mr. S. Warren had found M'Naughten a *sane* man, and had been informed that he had given no evidence of derangement of mind from the moment he was transferred from Newgate to Bethlem Hospital, then our opinion of his view of the trial would have been somewhat modified, and we should not have considered it our duty to make his paper the subject of a special article in our journal. We quote, without any further remarks of our own, Mr. Warren's graphic and interesting account of his visit to M'Naughten:—

"M'Naughten was standing in the courtyard, dressed in the costume of the place, (a pepper-and-salt jacket and corduroy trousers,) with his hat on, knitting. He looks about forty years old, and in perfect health. His features are regular, and their expression is mild and prepossessing. His manner is tranquil. Usually he wears his hat somewhat slouched over his eyes, and sidles slowly away from any one approaching him, as if anxious to escape observation; but on this occasion he at once entered into conversation with our companion, calmly and cheerfully, and afforded us a full opportunity of watching him. Had we seen him casually elsewhere, and as a stranger, we should have thought his countenance indicative of a certain sort of cheerful quiet humour, especially while he was speaking; but to us it seemed certainly to exhibit a feeble intellect, shown chiefly by a faint flickering smile, even when he was speaking on the gravest subjects.—When asked what had brought him where he was, he replied, '*Fate.*' 'And what is fate?' 'The will of God, or perhaps'—he added quickly, 'of the devil—or it may be of both?' and he half closed his eyes and smiled.—[The reader will bear in mind what was deposed at the trial, as to his infidel tendencies.]—When told that Sir Robert Peel was dead, he betrayed no emotion, nor exhibited the slightest interest. 'One should have thought that, considering what has happened, you would have felt some interest in that gentleman.' He looked rather quickly at the speaker, and said, calmly, with a faint smile, 'It is quite useless to talk to me on *that* subject: you know quite well I have long and long ago made up my mind never to say one word about it. I never have, and I never will; and so it would be quite childish to put any questions.'* . . . . 'How are you, M'Naughten?'—He slightly sighed, and said, 'I am very uncomfortable. I am very ill-used here; there is somebody [or something] always using me ill here. It is really too bad! I have spoken about it many, many times; but it is quite useless. I wish I could get away from this place! If I could just get out of this place, and go back to Glasgow, my native place, it is all I would ask for: I should be quite well there! I shall never be well or happy *here*, for there is always some one ill-using me here.' 'Well, but what do they do to you?' 'Oh,' shaking his head, and smiling, 'they are always doing it; really it is too bad.'—'Who are they?' 'Oh, I am always being ill-used here! My only wish now is, to get away from this place! If I could only once get to Glasgow, my native place!' This is the continual burthen of his song. It is needless to say that his complaints are altogether unfounded; he is treated with the utmost kindness consistent with his situation; and, as he has never exhibited violence nor ill-behaviour, it has never been necessary to resort to personal coercion, with one exception. Two or three years ago, he took it into his head that, as he could not get away, he would starve himself; and he persevered for such a length of time in refusing all kind of food that he began to lose flesh fast. At length he was told by the physician that, since he would not eat voluntarily, he must be made to eat; and it was actually necessary to feed him for a considerable time mechanically, by means of the stomach-pump. Under this treatment he presently regained his flesh, in spite—as it were—of himself; and at length suffered himself to be laughed out of his obstinacy, and has ever since taken his food voluntarily. He seemed himself to be tickled by a sense of the absurdity of which he was guilty. Not a doubt of his complete insanity was entertained by my acute companion, who has devoted much observation to the case. Shortly after we had quitted him, and were out of his sight, he put away his knitting, placed his hands in his jacket-pockets, and walked very rapidly to and fro, his face bent on the ground; and he was apparently somewhat excited. Whatever may have been the state of M'Naughten at the time to which our inquiries have been directed in this article, we entertain little, if any doubt, that he is now in an imbecile condition.'

As illustrative of some remarks we made in a previous number of this journal (Oct. 1850), when commenting on the trial of Pate, we extract also Mr. Warren's description of a conversation he had with Oxford, another criminal lunatic:—

"Oxford was in another part of the building, standing alone, at the extremity of a long corridor, gazing through a heavily-grated window, towards the new House of Parliament. His hat was on; he was

* This he has always said, and has adhered to his resolution.

dressed like M'Naughten, and his jacket was buttoned. We scarcely recognised him, owing to the change of his dress. He is fond of attracting the notice of anybody; and conversed about himself and his offence in the most calm and rational manner conceivable. He has lost much of his hair—a circumstance which he appeared somewhat to regret—for the front of his head is bald; but he looks no older than his real age, thirty. He is mortally weary of his confinement, and says he has been terribly punished for 'his foolish act.' '*Foolish!*' we exclaimed—'is *that* all you can say of your attempt to shoot her Majesty?' He smiled, and said, "Oh, sir, *I* never attempted to shoot her; I never thought of such a thing. I aimed at the carriage-panels only.' 'Then why did you put balls in your pistols?' 'I never did,' he replied quickly. 'I never dreamed of such a thing. There were no balls.' 'Oh, then you have not heard of the discovery that has just been made—eh?' 'Discovery—what?' 'The bullets.' 'Oh, there have been more found than ever *I* used at least; for I assure you I never used any!' 'What made you do what you did?' 'Oh, I was a fool; it was just to get myself talked about, and kick up a dust. *A good horsewhipping was what I wanted*,' he added, with a faint sigh. These were his very words. 'Should you have done it, if you had thought of coming *here?*' 'No, indeed, I should not; it has been a severe punishment! I dare say public opinion says nothing about me now; I dare say it thinks I have got what I very well deserve—and perhaps I have; but possibly if I were put quietly out of the way, and sent abroad somewhere, public opinion might take no notice of it.' He has taught himself French, Italian, and German, of which he has a fair knowledge. He also used to draw a little, and began to write a novel; but it proved a sorry affair, and, being discouraged, he threw it up. 'Do you recollect hearing the condemned sermon preached to Courvoisier?' 'Oh, yes, very well. It was a most excellent sermon.' 'Did Courvoisier seem to attend to it?' 'Oh, yes, very much; and he seemed very much affected. It was certainly a very appropriate sermon; I liked it much.' 'Did not you think that it might soon be your fate to sit where he was?' 'What, in the condemned seat.' 'Yes.' 'Oh, no; that never occurred to me. I never expected to be condemned for high treason. Some gentleman—I forget who he was—said I should be transported for fourteen years. I thought that was the worst they could do to me; for I knew I had never meant to do any harm, nor tried to do it.' 'Yes; but the judge and jury thought very differently.' 'Oh, I was very fairly tried; but I never expected to be brought in *mad.* I was quite surprised at *that,* for I knew I was not mad, and I wondered how they were going to prove it.' We asked him if he had ever seen *us;* to which he replied, gazing steadily, 'Yes. I think I have—either at the Privy Council, or in Newgate Chapel.' 'Where did you sit on the Sunday when the condemned sermon was preached to Courvoisier?' 'I sate on the steps near the altar.' 'How were you dressed?' 'Oh, a blue surtout, with velvet collar;' and he proceeded to describe his dress almost exactly as we have described it at the commencement of the article. He exhibits considerable cleverness: whatever he does, whether in playing at fives, or working, (*e. g.* making gloves, &c.) he does far better than any one else, and shows considerable tact and energy in setting his companions to work, and superintending them. He admits that he committed a very great offence in having done anything to alarm the Queen, and attributes it entirely to a mischievous and foolish love of notoriety. He said, 'I thought it would set everybody talking and wondering;' but 'never dreamed of what would have come of it—least of all that I was to be shut up all my life in *this* place.' . . . . 'That list of conspirators, and letters from them, that were found in your lodgings—were they not real?' 'Oh, no,' he replied, with rather an anxious smile, 'all mere sham—only nonsense! There was never anything of the sort!' 'Then, why did you do it?' 'It was only the folly of a boy; I wasn't nineteen then—it was very silly, no doubt.' 'And their swords and dresses, and so forth—eh?' 'Entirely nonsense! It was a very absurd joke. I did not think it would come out so serious. I did not *appreciate* the consequences, or I never would have done it.' The word 'appreciate' he used with a very marked emphasis.

"We entertain no doubt whatever of his perfect sanity; *and, if so,* as his crime was great, so his punishment is fearful."

---

